# Disparities in Heart Failure Deaths among Patients with Cirrhosis

**DOI:** 10.3390/jcm13206153

**Published:** 2024-10-16

**Authors:** Benjamin Grobman, Arian Mansur, Christine Y. Lu

**Affiliations:** 1 Harvard Medical School, 25 Shattuck Street, Boston, MA 02115, USA; 2 Department of Population Medicine, Harvard Pilgrim Health Care Institute, Boston, MA 02215, USA; 3 School of Pharmacy, Faculty of Medicine and Health, The University of Sydney, Sydney, NSW 2050, Australia; 4 Kolling Institute, Faculty of Medicine and Health, The University of Sydney, The Northern Sydney Local Health District, Sydney, NSW 2065, Australia

**Keywords:** heart failure, cirrhosis, disparities

## Abstract

**Background:** Heart failure deaths have increased in recent years in the United States and are projected to continue to increase in the future. Rates of liver disease and cirrhosis have similarly increased in the United States. Patients with cirrhosis are at an elevated risk of heart failure with a worsened prognosis. As such, investigations of the epidemiology of these comorbid conditions are important. **Methods:** We obtained data on heart failure deaths among people with cirrhosis in the United States from 1999 to 2020 from the Centers for Disease Control Wide-ranging Online Data for Epidemiologic Research multiple cause of death database. Rates were analyzed for the population as a whole and for demographic subgroups. **Results:** From 1999 to 2020, there were 7424 cirrhosis-related heart failure deaths. Rates were higher among Black (AAMR ratio = 1.288, 95% CI: 1.282–1.295) and Asian people (AAMR ratio = 3.310, 95% CI: 3.297–3.323) compared to White people. Rates were also higher in rural areas than in urban areas (AAMR ratio = 1.266, 95% CI: 1.261–1.271). Rates increased over time across demographic subgroups. **Conclusions:** People with cirrhosis are at an elevated risk of heart failure death compared to the general population. Rates were particularly elevated in Asian people, Black people, males, and people living in rural areas. These data indicate a significant and previously underappreciated disease burden. Clinicians taking care of cirrhosis patients should be aware of the risk of heart failure and should collaborate with cardiac specialists as needed.

## 1. Introduction

Heart failure is a rising issue both in the United States and around the world. Around 6.7 million Americans over the age of 20 have heart failure, with an expected increase in prevalence of 1.8 million by the year 2030 [1]. The prognosis for patients with heart failure remains poor. Not only is heart failure a secondary cause of death for many people, but 300,000 yearly deaths are primarily due to heart failure [2]. Each year, heart failure costs the United States an estimated $179.5 billion [3]. In recent decades, the lifetime risk of heart failure in the United States has similarly increased from 1 in 5 to 1 in 4 [1]. The causes of this increase are multiple: in part, heart failure prevalence has increased due to improved treatment leading to an increased number of people living with chronic heart failure. Between the years 1970–1979 and 2000–2009, the 5-year heart failure survival rate improved from 29% to 60% [4]. Additionally, as the average age of the US population continues to rise, the number of people living with heart failure is increasing [5]. Furthermore, while the prevalence of some risk factors, such as hyperlipidemia and smoking, have declined, others, such as diabetes and obesity, have increased, further contributing to the ongoing epidemic of heart failure [5].

Moreover, the risk of heart failure is not evenly distributed among populations in the United States: Black and Hispanic patients in the United States have a higher prevalence of heart failure compared to people of other demographics, with particularly elevated incidence among younger Black patients [6]. The reasons for these disparities are multifactorial, but are in large part due to higher rates of risk factors such as diabetes, obesity, and hypertension among minority patients [6].

Rates of liver disease and cirrhosis in the United States have also significantly increased in recent years. Between 1990 and 2019, deaths due to cirrhosis increased by 87%, and by 2019 cirrhosis was the 11th leading cause of death in the United States [7]. This rapid increase is due to multiple factors, but a particularly significant cause is an increase in metabolic dysfunction-associated fatty liver disease (MAFLD) second to rising rates of obesity [7]. For example, MAFLD prevalence is particularly high among Latino Americans [7]. There are also significant disparities in the cirrhosis deaths, with recent increases in the White population while rates have decreased in other groups [7]. These changes represent differences in the epidemiology of cirrhosis over time. Historically, the primary cause of cirrhosis of the liver in the United States was the hepatitis C virus [8]. However, in recent years, control of hepatitis has improved in the United States, while cirrhosis due to metabolic dysfunction and alcohol consumption has continued to rise [8].

Cirrhosis has many complications and patients with cirrhosis are at an elevated risk of multiple diseases. Chief among these is hepatocellular carcinoma (HCC): globally, in 2020 there were over 900,000 people diagnosed with hepatocellular carcinoma and over 800,000 deaths. In the coming years, these rates are expected to rise globally with the potential for over 1 million HCC deaths by the year 2040 [9]. While death due to cirrhosis itself and complications such as HCC are the most common diseases attributed to cirrhosis, there is a significant burden of heart failure among those with cirrhosis [10]. Patients with cirrhosis are at an elevated risk of heart failure and vice versa, and those with concomitant heart failure and cirrhosis have a worse prognosis than those with either disease individually [10]. Up to 50% of patients with cirrhosis have cirrhotic cardiomyopathy, characterized by impaired systolic and diastolic cardiac function [10]. Prior studies in smaller cohorts have shown that patients with end-stage liver disease are at a higher risk of incident heart failure and that this risk persists independent of shared risk factors [11]. Despite this known interplay, prior research has not examined these intersecting risks on a national scale. Additionally, there is minimal information on disparities in deaths among people with both cirrhosis and heart failure: this question is particularly important given the multiple manifestations of the social determinants of health within these diseases.

This study examines rates of cirrhosis-related heart failure deaths overall and by race, gender, age, and geography, as well as how these disparities have changed over time. Findings can inform tailored treatment and public health interventions in disparate populations to reduce heart failure mortality among people with cirrhosis.

## 2. Materials and Methods

We analyzed the Centers for Disease Control and Prevention Wide-ranging Online Data for Epidemiologic Research (CDC WONDER) multiple cause of death database for deaths among people with heart failure with comorbid cirrhosis [12]. Data in CDC WONDER are obtained from national records of death certificates in the United States [12]. Each death certificate contains one primary cause of death and up to 20 multiple causes of death [12]. We analyzed deaths in the United States with heart failure as the primary cause of death (ICD-10 code I50) and chronic liver disease and cirrhosis listed as a multiple cause of death (ICD-10 codes K70, K73–74) among people aged 45 and older. In prior studies, less than 10% of patients with cirrhosis are under the age of 45 [13]. We also examined rates of heart failure death among people with diabetes mellitus (ICD-10 codes E10–E14) and cirrhosis as multiple causes of death as these conditions are linked and diabetes is a major risk factor for heart failure and as such may confound this relationship [14,15].

We obtained information on death certificates from the CDC WONDER database. To calculate the age-adjusted mortality rates for heart failure and cirrhosis, we used a multi-step method. First, we obtained the number of deaths with heart failure as the primary cause of death and cirrhosis as a multiple cause of death (regardless of the primary cause of death) for each 10-year age group ranging from 45–54 to 85+ (45–54 years, 55–64 years, 65–74 years, 75–84 years, and 85+). In order to estimate the death rate per 100,000 from heart failure among those with cirrhosis, we divided the number of deaths in the 10-year age group where both heart failure was the primary cause of death and cirrhosis was a multiple cause by the total number of cirrhosis deaths as a multiple cause among the corresponding 10-year age group. To obtain the rate per 100,000, we then multiplied this number by 100,000. In order to estimate an age-adjusted rate, we then multiplied the rate by the corresponding age weight. Data on proper age-weighting to the 2000 U.S. census were obtained from the Centers from Disease Control [16]. To then obtain the age-adjusted death rate, we summed the weighted rate for each 10-year age group to create a single weighted rate for all age groups. We then repeated this process for each stratum individually. This process was completed using Microsoft Excel version 16.89.1. [17]. We also used the CDC WONDER database to obtain the number of deaths for each 10-year age group where diabetes was a multiple cause of death (regardless of the primary cause of death). Stratifications were race (Black, White, and Asian), ethnicity (non-Hispanic vs. Hispanic), region (Northeast, Midwest, South, and West), urban (defined as large central metropolitan area, large fringe metropolitan area, medium metropolitan area, or small metropolitan area), and rural (micropolitan or noncore region) [18].

We then calculated age-adjusted mortality rate ratios using R studio version 4.2.2 (USA). [19]. In order to calculate age-adjusted mortality rate (AAMR) ratios, we divided the age-adjusted death rates for the comparator group by the age-adjusted death rate for the reference group (e.g., the death rate among Black Americans divided by the death rate among White Americans). In order to obtain the 95% confidence intervals of the age-adjusted mortality rate ratios, we first calculated the standard error of the age-adjusted mortality rate ratio as the square root of the comparator standard error divided by the comparator rate squared plus the square root of the reference standard error divided by the reference rate squared. We then took the 95% confidence intervals to be the 97.5% and 2.5% boundaries surrounding the age-adjusted mortality rate rounded to three decimal points. Similar methods have been used in prior publications [18,20,21,22]. We tested for statistical significance using Z-tests. Analyses were conducted using Microsoft Excel version 16.89.1 (USA) and R studio version 4.2.2 (USA) [16,18]. Graphics were designed using ggplot2 [23]. This study used deidentified publicly available information and as such was exempt from IRB approval.

## 3. Results

**Figure 1:**
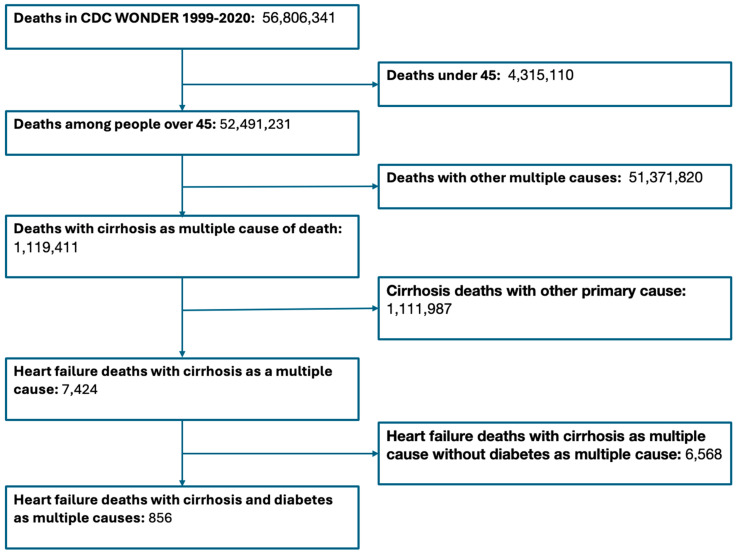
Study flowsheet.

During the study period (1999–2020), there were 1,415,533 deaths where heart failure was the primary cause of death. During this same period, there were 1,119,411 deaths among people with cirrhosis as a multiple cause of death regardless of primary cause of death (Figure 1). The overall heart failure death rate was 54.7 deaths per 100,000, while the death rate from cirrhosis as a multiple cause of death was 25.0 per 100,000 (Table 1). The rates of heart failure death were highest among Black Americans at 61.7 deaths per 100,000, followed by White Americans at 55.2 deaths per 100,000, followed by Asian Americans at 20.9 deaths per 10,000. For cirrhosis as a multiple cause of death, the death rates were the highest among White Americans at 43.5 deaths per 100,000, followed by Black Americans at 37.3 deaths per 100,000, followed by Asian Americans at 20.4 deaths per 100,000.

Death rates from heart failure were higher among Hispanic Americans (37.7 deaths per 100,000) compared to non-Hispanic Americans (23.8 deaths per 100,000). For cirrhosis as a multiple cause of death, death rates were higher among Hispanic Americans (65.0 deaths per 100,000) than among non-Hispanic Americans (40.0 deaths per 100,000).

Rates of heart failure death as a primary cause were highest in the Midwest at 62.1 deaths per 100,000, followed by the South at 60.6 deaths per 100,000, the Northeast at 49.2 deaths per 100,000, and the West at 41.6 deaths per 100,000. Rates of cirrhosis as a multiple cause of death were highest in the West at 50.1 deaths per 100,000, followed by the South at 44.8 deaths per 100,000. This was followed by the Midwest at 37.0 deaths per 100,000. The lowest death rate was in the Northeast at 33.8 deaths per 100,000.

The heart failure death rate was higher in rural areas at 69.2 deaths per 100,000, compared to 51.6 deaths per 100,000 in urban areas. For cirrhosis as a multiple cause of death, the death rate was higher in rural areas at 44.1 deaths per 100,000, compared to 41.5 deaths per 100,000 in urban areas.

Regarding sex, the age-adjusted heart failure death rate was higher among males at 60.4 deaths per 100,00 compared to 50.5 deaths per 100,000 among females. The death rate for cirrhosis as a multiple cause was higher among males at 58.6 compared to females at 27.6 deaths per 100,000.

Death rates for heart failure were highest between 2016 and 2020 at 58.4, followed by 1994–2004 at 57.0, 2005–2010 at 51.8, and 2011–2015 at 51.4. The death rate for cirrhosis as a multiple cause was highest between 2016 and 2020 at 48.5 deaths per 100,000, followed by 2011–2015 at 43.8 deaths per 100,000, followed by 2005–2010 at 38.2 deaths per 100,000, followed by 1999–2004 at 36.6 deaths per 100,000 (Table 1).

**Figure 2:**
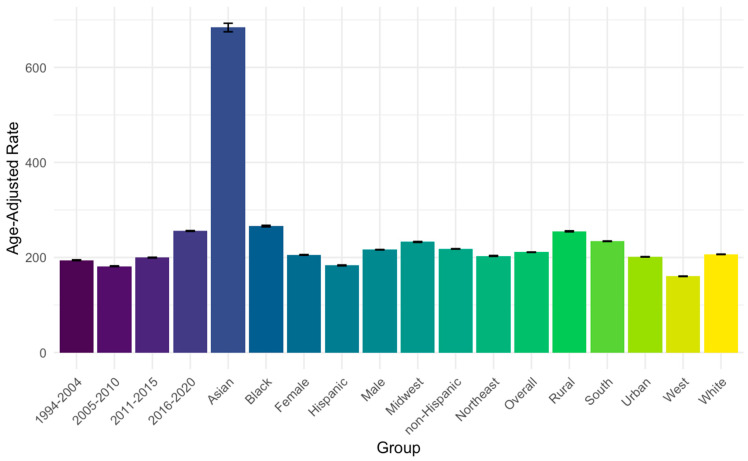
Rate of cirrhosis-related heart failure death among people with cirrhosis age 45+ 1999–2020 (rates per 100,000 people with cirrhosis) by demographic/regional subgroup. Legend: Bar height represents age-adjusted death rate for heart failure per 100,000 people with cirrhosis as a multiple cause of death. Error bars represent 95% confidence intervals for estimates.

During the study period, there were 7424 cirrhosis-related heart failure deaths (Table 2, Figure 2). The age-adjusted mortality rate for heart failure death among people with cirrhosis as a contributing cause of death was 211.1 deaths per 100,000 (95% CI: 210.7–211.5). There were 856 deaths among people with heart failure and comorbid diabetes and cirrhosis (AAMR = 233.9, 95% CI: 232.5–235.3).

During the study period, there were 768 cirrhosis-associated heart failure deaths among Black Americans, 129 deaths among Asian Americans, and 6449 deaths among White Americans. The rate of cirrhosis-associated heart failure deaths was higher among Black (AAMR ratio = 1.288, 95% CI: 1.282–1.295) and Asian people (AAMR ratio = 3.310, 95% CI: 3.297–3.323) compared to White people (Figure 2; Table 2). Compared to non-Hispanic people, cirrhosis-associated heart failure deaths were lower among Hispanic people (AAMR ratio = 0.841, 95% CI: 0.835–0.847).

During the period 2016–2020, the rate of cirrhosis-associated heart failure deaths was higher among both Black (AAMR ratio = 1.525, 95% CI: 1.508–1.542) and White people (AAMR ratio = 1.301, 95% CI: 1.295–1.307) as compared to the period 1999–2004 (Table 3). The differences in rates of cirrhosis-related heart disease deaths between White and Black people widened over time from 40.3 per 100,000 in 1999–2004 to 104.1 in 2016–2020 (Table 4).

**Figure 3:**
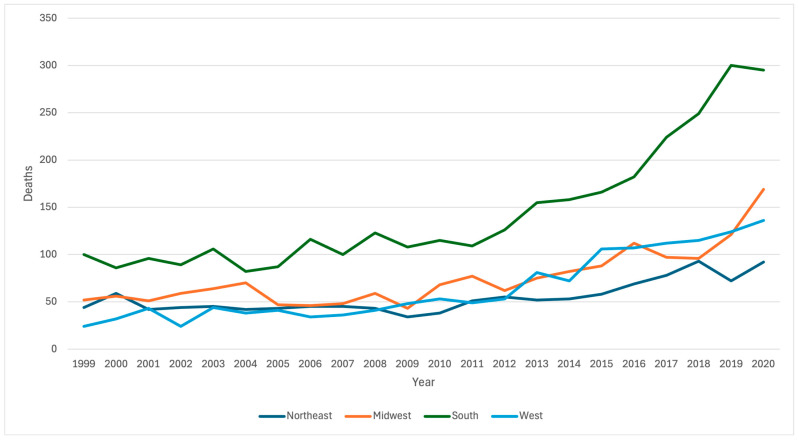
Deaths from heart failure among people aged 45+ with cirrhosis as a multiple cause of death in 1999–2020 by year and region.

During the study period, there were 1197 cirrhosis-associated heart failure deaths in the Northeast, 1642 deaths in the Midwest, 3172 deaths in the South, and 1413 deaths in the West. Rates of cirrhosis-associated heart failure deaths were higher in the Midwest (AAMR ratio = 1.145, 95% CI: 1.138–1.151) and South (AAMR ratio = 1.152, 95% CI: 1.146–1.158) compared to the Northeast but were lower in the West (AAMR ratio = 0.789, 95% CI: 0.783–0.795) (Table 2). Between 1999–2004 and 2016–2020, the rates of cirrhosis-associated heart failure deaths increased in all regions: the AAMR ratio was 1.225 (95% CI 1.211–1.238) for people living in the Northeast, 1.147 (95% CI: 1.135–1.159) for people living in the Midwest, 1.726 (95% CI: 1.715–1.737) for people living in the West, and 1.292 (95% CI: 1.283–1.300) for people living in the South (Table 3, Figure 3).

During the study period, there were 5848 cirrhosis-associated heart failure deaths in urban areas and 1576 deaths in rural areas. Cirrhosis-associated heart failure deaths in people living in rural areas were higher than those in urban areas (AAMR ratio = 1.266, 95% CI: 1.261–1.271) (Table 2). Between 1999–2004 and 2016–2020, the rate of cirrhosis-associated heart failure deaths among people living in urban and rural areas increased, with AAMR ratios of 1.361 (95% CI: 1.3556–1.367) and 1.149 (95% CI: 1.136–1.162), respectively (Table 3). The differences in the rates of cirrhosis-related heart disease deaths between people living in urban and rural areas narrowed over time from 78.1 per 100,000 in 1999–2004 to 51.4 in 2016–2020 (Table 4).

During the study period, there were 2910 cirrhosis-associated heart failure deaths among females and 4514 deaths among males. The rate of cirrhosis-associated heart failure deaths was higher among males than among females (AAMR ratio = 1.053, 95% CI: 1.050–1.057; Table 2). Rates of cirrhosis-associated heart failure deaths increased in males between 1999–2004 and 2016–2020 (AAMR ratio = 1.408, 95% CI: 1.401–1.414) and in females (AAMR ratio = 1.197, 95% CI: 1.188–1.206) (Table 3). The differences in rates of cirrhosis-related heart disease deaths between males and females widened over time from 8.9 per 100,000 in 1999–2004 to 29.5 in 2016–2020 (Table 4).

**Figure 4:**
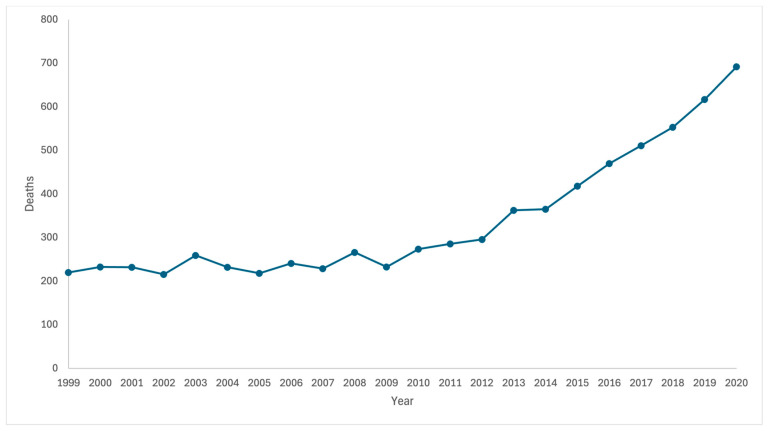
Deaths from heart failure among people aged 45+ with cirrhosis as a multiple cause of death in 1999–2020 by year.

During the period examined, there were 1392 cirrhosis-associated heart failure deaths between 1999 and 2004, 1461 deaths between 2005 and 2010, 1728 deaths between 2011 and 2015, and 2853 deaths between 2016 and 2020. During the period 1999–2004, the AAMR for cirrhosis-related heart failure deaths increased from 194.4 (95% CI: 193.6–195.2) to 255.9 (95% CI: 255.0–256.7) in 2016–2020, with an AAMR ratio of 1.317 (95% CI: 1.311–1.322) (Figure 4).

## 4. Discussion

In the present study, using national death certificate data, we found that cirrhosis-related heart failure deaths increased over time. We also found that there are significant disparities in this disease burden along lines of geography and sex, with particularly high rates among Asian Americans and people in rural areas.

With regard to race, Black Americans had a significantly higher risk of cirrhosis-associated heart failure death when compared to White Americans. This finding is consistent with prior analyses that showed that Black individuals with cirrhosis or liver disease have higher rates of all-cause mortality and non-liver-related mortality when compared to White individuals [24,25]. These reasons for this observed disparity are likely multifactorial. Prior studies have shown Black Americans with chronic liver disease and cirrhosis have higher mortality and lower rates of transplant than White Americans [24]. Moreover, both Black and White Americans experienced an increase in the rate of cirrhosis-associated heart failure deaths in recent years (2016–2020) when compared to prior years (1999–2004), suggesting an increasing burden of cirrhosis on heart failure deaths. This finding is consistent with prior analyses showing that liver disease is associated with increased cardiovascular burden [26,27]. We particularly found that Asian Americans with cirrhosis had a particularly high risk of heart failure mortality. This is a novel finding, and one which requires further investigation given that Asian Americans have lower overall rates of heart failure mortality and lower rates of cirrhosis mortality [6,7]. However, some prior research has suggested that people of East Asian descent may be susceptible to heart failure and metabolic-associated fatty liver disease at lower BMIs than their Caucasian counterparts; as such, it is possible that patients of Asian descent with these comorbid conditions may have worse outcomes [28,29]. We also found that Hispanic patients with cirrhosis had a lower rate of heart failure mortality compared to their non-Hispanic counterparts. This aligns with previous research showing that Hispanic patients have lower rates of death from heart failure [6]. There is an extensive prior body of research which has shown that despite adverse socioeconomic and risk factor burdens, Hispanic patients have a lower mortality burden across a range of conditions [30]. This has been observed in the setting of cirrhosis, where Hispanic patients have higher rates of non-alcoholic fatty liver disease despite lower socioeconomic status [31]. Despite this well-established pattern, no clear single reason has been established. However, theories include the healthy migrant hypothesis in which Hispanic immigrants to the United States are healthier than the average population, as well as increased social support due to cultural factors [30,31].

With regard to US census region, people living in the South had the highest risk of cirrhosis-associated heart failure death, followed by the Midwest, Northeast, and West. However, we found the largest increase in deaths in the West (AAMR ratio 1.726), followed by the South (AAMR ratio 1.292), Northeast (AAMR ratio 1.225), and Midwest (AAMR ratio 1.147). Our findings are consistent with prior research that shows that heart failure deaths are highest in the South and Midwest [32,33]. These findings are reflected by multiple worse health indicators in this region. Average socioeconomic status in the Southern United States is the lowest of all major regions in the United States [34]. Prior research has shown that low socioeconomic status is a predictor of incident heart failure. Additionally, among patients with heart failure, lower income and lower socioeconomic status are associated with higher mortality [35]. Prior research has also shown that the ‘southern diet score’, a measure of how closely a diet adheres to that which is classically found in the Southern United States, is one of the strongest predictors of higher metabolic dysfunction. The Southern diet is one with high consumption of fried food, eggs, processed and organ meat, and sweetened beverages [36]. Those who eat diets which adhere to this pattern have higher rates of incident heart failure and have a higher risk of obesity [36,37]. Prior research has also shown that people in the Midwest have poorer diets than those in the Northeast and West, with lower intake of vegetables, whole grains, and fruits [38].

With regard to urbanicity, individuals living in rural areas had a higher risk of death from cirrhosis-associated heart failure when compared to those living in urban areas. This is consistent with prior research showing increased risk of death in rural areas for those with heart failure and those with cirrhosis [1,39]. Interestingly, those in urban areas (AAMR ratio 1.361) experienced a larger relative increase in death rate over time than those in rural areas (AAMR ratio 1.149). In the United States in general, rural areas have significantly worse health outcomes than urban areas. This has been seen across multiple conditions: for example, a previous study on patients with chronic obstructive pulmonary disease (COPD) showed that the prevalence of COPD was higher in rural than in urban areas; rural residents with COPD had lower levels of education, income, and insurance, and were less able to afford healthcare [40]. Research on cardiovascular diseases has shown similar patterns: patients treated for heart failure, heart attack, and stroke in rural areas have higher mortality than their urban counterparts [41]. The prevalence of heart failure is higher in rural areas than in urban areas, even when adjusting for cardiovascular risk factors, socioeconomic status, and behavioral risk factors, with particularly large disparities seen among minorities [42]. Similar patterns have been seen in investigations of disparities in deaths from cirrhosis. For example, a previous study using the CDC WONDER database showed that there are higher rates of cirrhosis in rural areas than in urban areas, and that these disparities have widened in recent decades [39]. Prior studies have shown that people in rural areas are more likely to report past-year alcohol use disorder, and to report lower rates of alcohol abstinence than their urban counterparts [43].

With regard to sex, we found that males had a higher risk of cirrhosis-associated heart failure death when compared to females, and this disparity increased throughout the study period. This finding is consistent with prior analyses on heart failure [1,33]. Men have a higher risk of heart disease and its complications for multiple reasons. Men generally have higher rates than women for many of the most common cardiovascular risk factors, notably high blood pressure, high cholesterol, and smoking. By contrast, women have higher rates of obesity [44]. Additionally, among those with risk factors, rates of control of diabetes and hypertension are lower among men than among women [44]. In addition to these elevated rates of risk factors for heart disease and heart failure, men have higher rates of cirrhosis than women [45]. This is largely due to lower rates of hepatitis C and hepatitis B in women, as well as historically lower rates of alcohol consumption in women [46]. However, in recent years, the rates of hepatitis C- and hepatitis B-related liver disease have significantly decreased in the United States, while the rates of alcohol-related and metabolic-associated cirrhosis continue to rise [8]. In line with these findings, recent research has shown that among younger Americans, the rates of cirrhosis have risen faster in women than in men [47].

There are several strengths to this study. CDC WONDER contains information on deaths throughout the United States. Therefore, we were able to create a comprehensive analysis of death rates from heart failure and the contribution from cirrhosis. Furthermore, CDC WONDER also contains various variables, including geography, time period, demographics, and sex, which allowed us to draw insightful correlations.

The present study must be interpreted considering its limitations. Not all death certificates contain information on Hispanic ethnicity, and so our sample may be biased. Additionally, due to data suppression constraints we were not able to analyze deaths among Native Americans or to analyze trends over time among Asian or Hispanic patients. Additionally, the CDC WONDER database contains demographic information but does not contain information on individual socioeconomic status or healthcare access, which may be important determinants of healthcare outcomes among this population. Due to a lack of individual-level data in CDC WONDER, we were not able to examine the association between combinations of risk factors and the risk of cirrhosis-associated heart failure death.

In this study, we found that people with cirrhosis are at an elevated risk of heart failure death compared to the general population and this rate increased over time. Additionally, we found that rates of cirrhosis-related heart failure death were higher in Asian people, Black people, males, and people living in rural areas. Particular attention is required to ameliorate cirrhosis and improve the provision of cardiovascular healthcare to individuals of these demographic groups. Through the insights into the epidemiology and disparities in these comorbid conditions, policymakers and clinicians can better evaluate patients and design interventions to reduce cardiovascular risk among those with cirrhosis.

## Figures and Tables

**Table 1:** US population heart failure deaths and underlying cirrhosis-related deaths (1999–2020) among adults aged 45.

Category		Heart Failure as Primary Cause of Death	Cirrhosis as Multiple Cause of Death (Regardless of Primary Cause of Death)	Heart Failure Death Rate	Rate of Cirrhosis as a Multiple Cause of Death
Overall		1,415,533	1,119,411	54.7	42.2
Race					
	Black	139,073	108,240	61.7	37.3
	White	1,252,933	968,619	55.2	43.5
	Asian	18,373	22,304	20.9	20.4
Ethnicity					
	Hispanic	52,041	151,432	37.7	65.0
	Non-Hispanic	1,361,099	963,785	23.8	40.0
Region					
	Northeast	262,327	170,249	49.2	33.8
	Midwest	371,686	217,435	62.1	37.0
	South	554,864	439,767	60.6	44.8
	West	226,656	291,960	41.6	50.1
Urbanization					
	Urban	1,097,013	810,721	51.6	41.5
	Rural	318,520	308,690	69.2	44.1
Sex					
	Female	817,396	393,513	50.5	27.6
	Male	598,137	725,898	60.4	58.6
Year					
	1999–2004	335,850	220,543	57.0	36.6
	2005–2010	343,911	265,792	51.8	38.2
	2011–2015	325,075	286,259	51.4	43.8
	2016–2020	410,697	346,817	58.4	48.5

**Table 2:** Cirrhosis-related heart failure deaths per 100,000 individuals with cirrhosis aged 45+.

Category		Cirrhosis-Associated Heart Failure Deaths	AAMR Ratio	*p*-Value	
Overall		7424	211.1 (210.7–211.5)		
With Comorbid Diabetes		856	233.9 (232.5–235.3)		
Race					
	White	6449	206.6 (206.2–207.0)	ref	ref
	Black	768	266.2 (264.6–267.8)	1.288 (1.282–1.295)	<0.0001
	Asian	129	683.8 (674.9–692.9)	3.310 (3.297–3.323)	<0.0001
Ethnicity					
	Non-Hispanic	6668	218.1 (217.7–218.6)	ref	ref
	Hispanic	739	183.4 (182.4–184.4)	0.841 (0.835–0.847)	<0.0001
Region					
	Northeast	1197	203.3 (202.4–204.3)	ref	ref
	Midwest	1642	232.7 (231.8–233.7)	1.145 (1.138–1.151)	<0.0001
	South	3172	234.2 (233.6–234.9)	1.152 (1.146–1.158)	<0.0001
	West	1413	160.4 (159.9–161.0)	0.789 (0.783–0.795)	<0.0001
Urbanization					
	Urban	5848	201.5 (201.0–201.9)	ref	ref
	Rural	1576	255.0 (253.9–256.2)	1.266 (1.261–1.271)	<0.0001
Sex					
	Female	2910	205.4 (204.8–206.0)	ref	ref
	Male	4514	216.4 (215.9–216.9)	1.053 (1.050–1.057)	0.0004
Year					
	1999–2004	1392	194.4 (193.6–195.2)	ref	ref
	2005–2010	1461	181.8 (181.1–182.5)	0.935 (0.930–0.941)	<0.0001
	2011–2015	1728	199.9 (199.1–200.6)	1.028 (1.023–1.034)	<0.0001
	2016–2020	2853	255.9 (255.0–256.7)	1.317 (1.311–1.322)	<0.0001

AAMR: Age-adjusted mortality rate. Seventeen patients with cirrhosis-related heart failure deaths had their ethnicity listed as “not stated.” Age-adjusted mortality rates (AAMR) are standardized to the 2000 United States census using CDC WONDER data (1999–2020). AAMR ratio calculated as the AAMR of specific subgroups vs. reference group. No AAMR ratios were calculated for overall population and group with comorbid diabetes as there were no stratifications within these groups.

**Table 3:** Changes in cirrhosis-related heart failure death rate among adults with cirrhosis aged 45+ by time period.

Category		1999–2004	2016–2020	AAMR Ratio *	*p* Value
Race		Death rate per 100,000 (95% CI)	Death rate per 100,000 (95% CI)		
	White	191.5 (190.7–192.4)	249.2 (248.3–250.1)	1.301 (1.295–1.307)	<0.0001
	Black	231.8 (228.8–234.7)	353.3 (349.4–357.3)	1.525 (1.508–1.542)	<0.0001
Region					
	Northeast	198.4 (196.5–200.4)	243.0 (240.7–245.2)	1.225 (1.211–1.238)	<0.0001
	Midwest	237.2 (235.0–239.4)	272.1 (270.0–274.1)	1.147 (1.315–1.159)	<0.0001
	South	215.2 (213.8–216.7)	278.0 (276.6–279.5)	1.292 (1.283–1.300)	<0.0001
	West	122.5 (121.4–123.5)	211.4 (210.0–212.8)	1.726 (1.715–1.737)	<0.0001
Urbanization					
	Urban	181.2 (180.3–182.0)	246.6 (245.7–247.5)	1.361 (1.356–1.367)	<0.0001
	Rural	259.3 (256.6–261.9)	298.0 (295.7–300.3)	1.149 (1.136–1.162)	<0.0001
Sex					
	Female	199.3 (197.9–200.7)	238.5 (237.2–239.9)	1.197 (1.188–1.206)	<0.0001
	Male	190.4 (189.4–191.4)	268.0 (266.9–269.1)	1.408 (1.401–1.414)	<0.0001

* AAMR ratios calculated as demographic-specific ratios of 2016–2020 period: 1999–2004 period. AAMR: Age-adjusted mortality rate.

**Table 4:** Difference in cirrhosis-related heart failure death rate among adults with cirrhosis aged 45+ by time period.

Category		1994–2004	*p* Value	2016–2020	*p* Value
Race		Difference in Death Rate (Deaths per 100,000) ^1^		Difference in Death Rate (Deaths per 100,000)	
	Black vs. White	40.3	<0.0001	104.1	<0.0001
Region					
	Midwest vs. Northeast	38.8	<0.0001	29.1	<0.0001
	South vs. Northeast	16.8	<0.0001	35.0	<0.0001
	West vs. Northeast	−75.9	<0.0001	−31.6	<0.0001
Urbanization					
	Rural vs. Urban	78.1	<0.0001	51.4	<0.0001
Sex					
	Male vs. Female	−8.9	<0.0001	29.5	<0.0001

^1^ Difference in death rate calculated as age-adjusted death rate among first group vs. second group per 100,000 (e.g., Black/White) for given time period.

## Data Availability

The original data presented in the study are openly available at wonder.cdc.gov (accessed on 8 May 2024).
